# Self-paced graph memory for learner GPA prediction and it’s application in learner multiple evaluation

**DOI:** 10.1038/s41598-023-48690-5

**Published:** 2023-12-04

**Authors:** Yue Yun, Ruoqi Cao, Huan Dai, Yupei Zhang, Xuequn Shang

**Affiliations:** 1 School of Computer Science, Northwestern Polytechnical University, Xi’an, 710129 China; 2 Laboratory of Big Data Storage and Management, Ministry of Industry and Information Technology, Xi’an, 710129 China; 3 National Research University Higher School of Economics, Moscow, Russia 101000

**Keywords:** Computer science, Information technology

## Abstract

A scientific and rational evaluation of teaching is essential for personalized learning. In the current teaching assessment model that solely relies on Grade Point Average (GPA), learners with different learning abilities may be classified as the same type of student. It is challenging to uncover the underlying logic behind different learning patterns when GPA scores are the same. To address the limitations of pure GPA evaluation, we propose a data-driven assessment strategy as a supplement to the current methodology. Firstly, we integrate self-paced learning and graph memory neural networks to develop a learning performance prediction model called the self-paced graph memory network. Secondly, inspired by outliers in linear regression, we use a t-test approach to identify those student samples whose loss values significantly differ from normal samples, indicating that these students have different inherent learning patterns/logic compared to the majority. We find that these learners’ GPA levels are distributed across different levels. Through analyzing the learning process data of learners with the same GPA level, we find that our data-driven strategy effectively addresses the shortcomings of the GPA evaluation model. Furthermore, we validate the rationality of our method for student data modeling through protein classification experiments and student performance prediction experiments, it ensuring the rationality and effectiveness of our method.

## Introduction

The advent of the digital era and the ensuing socioeconomic prosperity have brought about additional challenges for personalized education in meeting the technical demands of digital transformation. Additionally, the COVID-19 pandemic has further accelerated and expanded the ongoing digital transformation of educational institutions^1^. Unfortunately, the personalized education system has been slow to respond to these demands, resulting in a failure to fulfill its main objective of producing professionals equipped with the skills demanded by the labor market^2^. Moreover, the traditional GPA-based evaluation system has hindered the cultivation of a wide array of 21st-century skills, many of which cannot be adequately assessed through a single metric such as GPA (Grade Point Average)^3^. GPA is influenced by various complex factors, including grade inflation, which poses challenges for exceptional students to differentiate themselves based on GPA, and causes confusion and a lack of confidence among students with slightly lower GPA scores during their job search. Consequently, this can lead to inefficient allocation of human resources^4^.

Diversity in student assessment has been extensively researched and explored in personalized education, particularly through the lens of multiple intelligence theory. According to Gardner’s theory of multiple intelligences^5^, individuals possess multiple, relatively independent forms of information processing, each exhibiting unique aspects of their intelligence profile. This theory is gradually finding application in the teaching and learning process, recognizing that students learn differently based on various instructional design methods^6^. The aforementioned issues highlight the need for a personalized learning system^7^. A critical demand that arises is the ability to predict students’ learning performance, including early forecasting of final GPAs or scores. In the digital age, learning analytics can leverage data and algorithms to forecast student learning progress and outcomes, enabling proactive interventions^7^. Student learning performance prediction (SLPP) is an efficient, accurate, and necessary method of data mining in education. It serves as an invaluable tool for teaching assistance and curriculum selection, aligning with students’ learning potential and cultivation goals.

**Figure 1:**
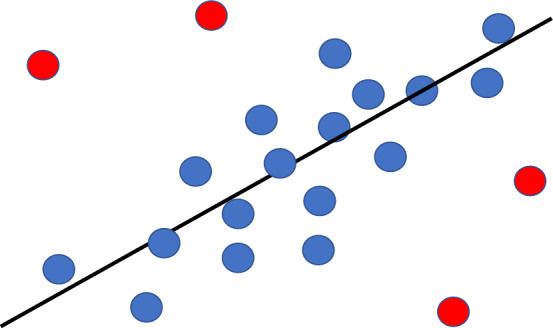
A simple example of resgression. The black line is the regression model that fits these samples. The red ones are outliers.

### Definition 1

*Distinctive Student* (DS) is the student whose learning pattern is clearly different from others. And, DSD is the abbreviation of Distinctive student detection. Specially, this definition is with respect to the data aspect.

Many methods have been utilized for the task of predicting student learning performance (SLP), encompassing decision trees^8^, k-nearest neighbors^9^, the naive Bayesian model^10^, and convolutional neural network^11^. Furthermore, researchers have employed graph representations of observed educational data^12,13^ in an effort to develop a suitable model that accurately predicts learners’ learning performance. While learning performance prediction is essential for personalized learning in personalized education, it is widely acknowledged that GPA alone is insufficient as an evaluation metric for students^14^. It is crucial to pay attention to students who do not conform to traditional GPA models and whose unique characteristics are not captured by such models. However, few researches focus on these points. To address this, we introduce the concept of distinctive students (DS), who form a small fraction of the overall student population but possess valuable educational insights. DSs exhibit various learning and lifestyle patterns, ranging from high GPAs with minimal exercise to low GPAs with extensive exercise. Correctly identifying DSs is crucial for providing individualized instruction and developing more effective student evaluation methods. Drawing inspiration from standard regression models, we present the following analogy:

In Fig. 1, the black line represents the best fit for the observed points, with blue and red points deviating from it. Similarly, GPA prediction models strive to fit all students’ data as closely as possible, but DSs deviate from these models. Unlike outliers in a regression model, DSs are excellent candidates for personalized education. For example, students with low GPAs and high levels of physical activity may require specific support from teachers.

We propose organizing the educational data into a graph and utilizing the Graph Memory Network (GMN) model^15^ to address these challenges. The GMN model is a significant variation of graph neural network (GNN) models^12,16,17^, and it has demonstrated remarkable performance in learning graph representations and prediction tasks. Therefore, in this article, we propose a robust model based on the GMN model (regression model in Fig. 1) for fitting all learners (sample points in Fig. 1) and completing the task of GPA prediction. Subsequently, we obtain a well-trained GPA prediction model and the loss values of all samples. Finally, similar to identifying outliers in Fig. 1, we complete the task of detecting abnormal students based on the obtained sequence of loss values.

However, according to the research of Khasahmadi^15^, the GMN model performs poorly on the Collab dataset. Analysis of the dataset reveals that the GMN model struggles to discover dense subgraphs and develop appropriate representations for graphs with dense communities. Unfortunately, graphs constructed with the educational data has a high edge-to-node ratio, resulting in dense communities. To address this weakness, we apply the self-paced learning (SPL) approach, a machine learning framework, to the GMN model. This leads to the development of the self-paced graph memory network (SPGMN), which aims to increase the efficiency of graph representation and enhance prediction performance. SPL in SPGMN assigns varying weights to training samples based on their difficulty, gradually introducing weighted samples into the training process from simpler to more complex ones. This method ensures smoother training for GMN models and helps them avoid local optimum solutions. Ultimately, SPL improves both the prediction performance of the GMN model and the detection performance of the DSD task.

In this paper, we focus on the student learning performance prediction (SLPP) and distinctive student detection (DSD) tasks. Then we aim to uncover insights hidden beneath the GPA metric by utilizing learning analytics and data mining to investigate the multiple evaluation in personalized education. To begin the study, we introduce self-paced learning (SPL) into graph memory networks (GMN) to improve the modeling of educational graphs. This leads to the proposal of a novel approach called the self-paced graph memory network (SPGMN) (i.e., Algorithm 1) . Furthermore, we suggest the SPGMN-DSD (distinctive student detection based on SPGMN) method to detect distinctive students using a combination of SLPP and DSD tasks (i.e., Algorithm 2). This approach aims to uncover additional interesting information that is not captured by the GPA measure, while also improving interpretability by recognizing distinct students based on a trained SPGMN model. Finally, we construct the data-driven evaluation methods based on the results of the DSD task.

The contributions of our work are listed as follows:

- Using the SPL framework, the representation efficiency of the GMN model is strengthened. As a result, for the SLPP problem, we suggested the SPGMN approach.
- The combination of the SLPP and DSD tasks enhances the interpretability of distinctive students. Consequently, this approach may provide more insightful findings than merely predicting accuracy. The SPGMN-DSD framework, which is a data-driven multiple evaluation approach, allows for the detection of distinctive students. This framework can help explain various phenomena that are not accounted for in the GPA evaluation framework, such as students with varying learning abilities achieving similar grades, and students with similar abilities receiving significantly different scores. Additionally, these findings can assist in identifying exceptionally gifted students (who achieve high grades with minimal practice) and students requiring immediate assistance (who have poor performance despite extensive practice).
- The SLPP task’s experimental findings validate the anticipated SPGMN method’s improvement. The experimental findings of the DSD task corroborate the concept that “GPA could not be the sole metric” from the standpoint of data science. Furthermore, the DSD task testing results reveal details behind unique students and give solid proof of personalized learning.

## Related works

### Student learning performance prediction

Student performance prediction is a core task in personalized education, and there have been many excellent related works in recent years. Zhang et al.^18^ summarized them as several methods, including traditional machine learning methods^19^, kernel methods^20^, collaborative filtering methods^21^, methods based on neural networks^22,23^, and so on. Among these methods, the neural network-based approach has attracted more extensive attention.

Nghe et al.^24^ investigated the decision tree and the Bayesian Network to predict the academic performance of undergraduates and postgraduates from two academic institutions. In their experiment, the accuracy of the DT is always 3–12% higher than the Bayesian Network. Psychology suggests that student evaluations are potentially influenced by their behaviors. Xu et al.^25^ categorized students into three groups based on their detailed records of learning activities on MOOC platforms: certification earning, video watching, and course sampling. The authors subsequently developed a predictor, using Support Vector Machines (SVM)^26^, to predict the attainment of certifications. Although these efforts have achieved significant results, the accuracy of predictions is not high. The introduction of deep neural networks has greatly improved the accuracy of predictions. Sorour et al.^27^ conducted experiments using the Latent Semantic Analysis (LSA) technique and an ANN model, achieving an average prediction accuracy of approximately 82.6%. Luo et al.^28^ utilized Word2Vec and an ANN to predict student grades in each lesson based on their comments. The experimental results demonstrated an 80% prediction rate for the 6 consecutive lessons and a final prediction rate of 94% for all 15 lessons.

### Graph memory network

#### Memory layer

**Figure 2:**
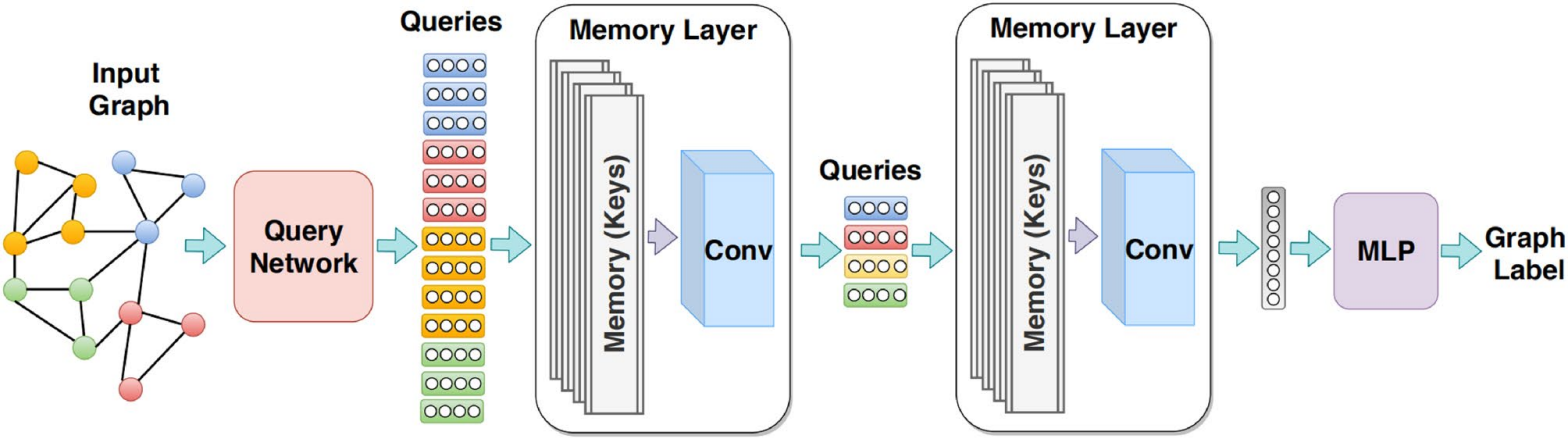
The workflow of graph memory network framework^15^.

Based on the work of graph neural networks, Khasahmadi et al. define a memory layer, denoted as $\mathscr{M}^l$, which is a parametric function that maps input query vectors of size $d_l$ from layer *l* in $\mathbb {R}^{n_l\times d_l}$ to output query vectors of size $d_{l+1}$ in $\mathbb {R}^{n_{l+1}\times d_{l+1}}$. This mapping reduces the number of query vectors from $n_l$ to $n_{l+1}$. The input queries represent the node representations of the input graph, while the output queries represent the node representations of the coarsened graph. The memory layer performs two tasks: pooling, which involves jointly coarsening the input nodes, and representation learning, which involves transforming their features. Fig. 2 illustrates a memory layer, which comprises multiple arrays of memory keys (referred to as multi-head memory) and a convolutional layer. Given that there are |*h*| memory heads, a shared input query is compared to all the keys in each head. This comparison generates |*h*| attention matrices, which are subsequently combined into a single attention matrix using the convolutional layer.

#### GMN architecture

A Graph Memory Network (GMN) consists of multiple memory layers stacked on top of a query network, which generates the initial query representations without any message passing. Similar to set neural networks and transformers, the nodes in GMN are regarded as a permutation-invariant set of representations. The role of the query network is to project the initial node features into a latent space that represents the initial query space.

The GMN process for graph classification involves two steps: (1) clustering the nodes of each graph and generating a representation vector for each graph, and (2) classifying these representation vectors. Consequently, the total loss of GMN is the combination of two loss functions: an unsupervised loss, denoted as $\mathscr{L}_{KL}^{(l)}$, and a supervised loss, denoted as $\mathscr{L}_{sup}$:

1 $$\begin{aligned} {\mathscr{L}\ }_{GMN}=\ \sum _{i\ =\ 1}^{n}\ell _i\ = \ \sum _{i\ =\ 1}^{N}{(\theta \mathscr{L}_{sup}\ +\ (1-\theta )\sum _{l\ =\ 1}^{L}\mathscr{L}_{KL}^{(l)})}, \end{aligned}$$

where *n* is the number of graphs, $\theta$ is a scalar weight, and *L* is the number of memory layers. For more details, please refer to this research paper^15^.

## Proposed work

### Motivation

The evolution of personalized education has given rise to diverse educational demands, rendering the traditional approach of evaluating students using only the GPA criterion inadequate to meet these varying needs. Additionally, it has led to the generation of a vast amount of educational data, including interaction data, learning environment data, and more. Leveraging this substantial volume of data allows for the successful implementation of multi-assessment strategies for students, which enables greater achievement and recognition compared to relying solely on the GPA metric. In light of this, we propose a data-driven multi-assessment method, which is built upon an improved GMN framework. Our method is founded on the GPA metric, with a primary focus on not only adopting relevant auxiliary indicators as supplements to GPA but also ensuring the accuracy of GPA prediction.

*Enhancing Learning Robustness* The task of predicting student learning performance is a crucial aspect of personalized learning, and the GMN framework holds the potential to achieve remarkable breakthroughs in graph representation and prediction tasks. As highlighted in^15^, GMN faces challenges when identifying graphs within densely connected communities, such as educational graphs. To enhance the resilience of the GMN framework, we adopt a two-step approach: initially learning from simpler samples to optimize parameters, and subsequently developing a more effective model capable of handling intricate data, including graphs with dense communities. Gradually incorporating increasingly complex samples during training fosters the generation of a more efficient model.

*Distinctive Student Detection* GPA is a widely used metric for evaluating students. However, according to educational experts, relying solely on a single GPA indicator is inadequate for evaluating students. And it is essential to prioritize students who deviate from typical GPA models, specifically, those who are considered distinctive.

Personalized learning aims to go beyond solely evaluating exam scores by assessing students’ individual learning progress^29^. Moreover, our aspiration to foster comprehensive student development drives us to examine the underlying principles of the GPA model and to prioritize the support and recognition of diverse learners. For instance, students who engage in extensive practice but struggle with their GPA may benefit from guidance from experienced professors to refine their learning strategies. Conversely, students who achieve high GPAs with minimal practice demonstrate their aptitude for grasping complex subjects. Thus, our research based on the distinctive student detection (DSD) task focuses on facts that the GPA statistic cannot capture. The indications concealed in these facts will be meticulously evaluated and developed as a supplement to the GPA metric in the task of student assessments.

**Figure 3:**
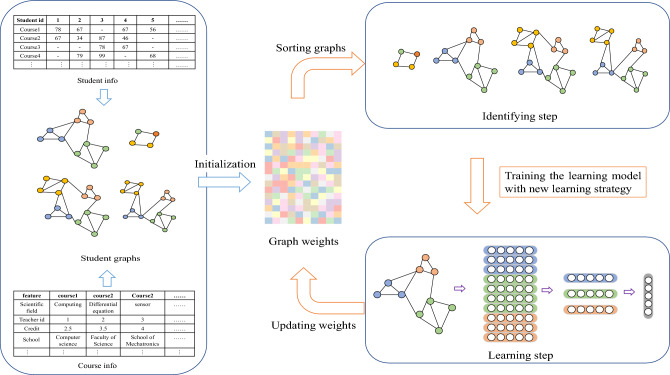
The framework of the proposed SPGMN. Note, the loop marked in orange arrows stopped when all the samples are involved into traning.

### Self-paced graph neural network model

The concept of self-paced learning (SPL) integrates a self-paced function and a pacing parameter into the learning objective, enabling the optimization of sample order and model parameters. The proposed SPGMN model’s schematic diagram is presented in Fig. 3. Weight allocation is utilized to gauge the difficulty of each sample, with gradual inclusion of these weighted samples in training, starting from simple to complex. In this study, we integrate a self-paced function into the learning objective of GMNs, allowing for joint learning of model parameters and latent weight variables. Specifically, the learning objective is formulated as follows:

2 $$\begin{aligned}  \min _{\textbf{w}, \textbf{v}} \mathbb {E}(\textbf{w}, \textbf{v}) = \sum _{i=1}^{n} \textbf{v}_i \cdot {\mathscr{L}\ }_{GMN} + f(\textbf{v}, \lambda ^{k}) = \sum _{i=1}^n \textbf{v}_i \cdot \ell _i\left( y_i, g\left( \textbf{x}_i, \textbf{w}\right) \right) +f\left( \textbf{v}, \lambda ^k\right) \end{aligned}$$

where ${\mathscr{L}\ }_{GMN}$ is the loss function of GMN refer to Eq. (1), $\textbf{w}$ is its parameter, $f(\textbf{v};k)$ is a dynamic self-paced function with respect to $v  \&  k$ , and $\lambda$ is the age of the SPGMN to control the learning pace, while **v** is designed to indicate which samples involved into training data. Consequently, $f(\textbf{v};k)$ is designed to explore a more optimal and robust learning strategy for subsequent processes. As $\lambda$ gradually increases, the training data incorporates samples in a progressively more complex manner (as selected by $\textbf{v}$), leading to the development of a more “mature” model.

### The optimization of SPGMN

For ease of understanding, we will refer to the $\mathscr{L}_{GMN}$ as $\ell _i$ in the following discussion. Additionally, we will use $\ell (\textbf{w})/\ell$ to represent $y \cdot \log \left( \frac{1}{g\left( \textbf{x}, \textbf{w}\right) }\right)$. In this context, we define a self-paced regularizer $f (\textbf{v}, \lambda ) = \lambda (\frac{1}{2}\textbf{v}^\textbf{2}\ -\ \textbf{v})$, and $\textbf{w}^k$ as the parameters of $g(\cdot )$ in the kth iteration. The learning process of SPGMN consists of two main steps: identifying step and learning step, as illustrated in Fig. 3. The identifying step involves searching for an optimized and robust learning strategy, where training samples are considered in increasing complexity from easy to complex. The learning step, on the other hand, focuses on learning the representation of the graph structure based on the selected strategy.

*Identifying step* To obtain more optimized learning strategy, we need to calculate $\textbf{v}^*(\ell _i({\textbf {w}}_k), \lambda )$ by solving the following problem:

3 $$\begin{aligned}  v_{i}^{*}\left( \ell _{i}\left( \textbf{w}^{k}\right) ,\lambda ^{k}\right) =\underset{\textbf{v} \in [0,1]}{{\text {argmin}}}v_{i}\ell _{i}\left( \textbf{w}^{k}\right) +\lambda (\frac{1}{2}\textbf{v}^\textbf{2}-\textbf{v}) = \left\{ \begin{array}{l} -\ell _i\left( \textbf{w}^k\right) /\lambda ^k+1, \ell _{i}\left( \textbf{w}^{k}\right) <\lambda ^{k} \\ 0, \ell _{i}\left( \textbf{w}^{k}\right) \ge \lambda ^{k}\end{array}\right. \end{aligned}$$

*Learning step* In this step, we already obtain the fixed $v_i^*$ and $\textbf{w}^k$ in the *k*-th iteration, thus, we obtain $\textbf{w}^{k+1}$ by calculating the following expression for the next iteration.

4 $$\begin{aligned}  \textbf{w}^{k+1}= \underset{\textbf{w}}{{\text {agrmin}}} \sum _{1}^{n} v_{i}^{*}\left( \ell _{i}\left( \textbf{w}^{k}\right) , \lambda \right) \ell _{i}\left( \textbf{w}^{k}\right) = \left\{ \begin{array}{c} \underset{\textbf{w}}{{\text {agrmin}}} \sum _{1}^{n} (\frac{-\ell _i\left( \textbf{w}^k\right) }{\lambda ^k}+1)\ell _i\left( \textbf{w}\right) , \ell _{i}\left( \textbf{w}^{k}\right) <\lambda ^{k} \\ 0, \quad \ell _{i}\left( \textbf{w}^{k}\right) \ge \lambda ^{k} \end{array}\right.  \end{aligned}$$

### Proposed algorithms

#### Algorithm of SPGMN

In Eqs. (3) and (4), the self-paced parameters, $\textbf{v}$ and $\textbf{w}$, are optimized iteratively through step-wise updates until the convergence of the objective function or exhaustion of the samples. To determine the increasing pace, $\lambda ^k$^30^, we predefine a sequence $N = \{n_1, n_2, n_3, ..., n_{\text {max}}\}$, where $n_i < n_j$ for $i < j$, representing the number of selected samples in the training process. Each $n_t$ indicates the number of samples to be selected in the *t*-th iteration, and $n_{\text {max}} = n$ implies the involvement of all samples in training. In the *t*-th iteration, we determine $L_{s}$ by sorting the loss function, ${\mathscr{L}_{\text {GMN}}}$, in ascending order and selecting the $n_t$-th loss value as the estimate of $\lambda ^k$. Therefore, $\lambda ^t$ can be represented as follows:

5 $$\begin{aligned}  \lambda ^t = {L_{s}}_{n_t}, \end{aligned}$$

where $L_{s}$ is obtained by sorting the loss ${\mathscr{L}\ }_{\text {GMN}}$ in ascending order.

We list the detailed steps in Algorithm 1 for a better understanding of the training process.

**Algorithm 1:**
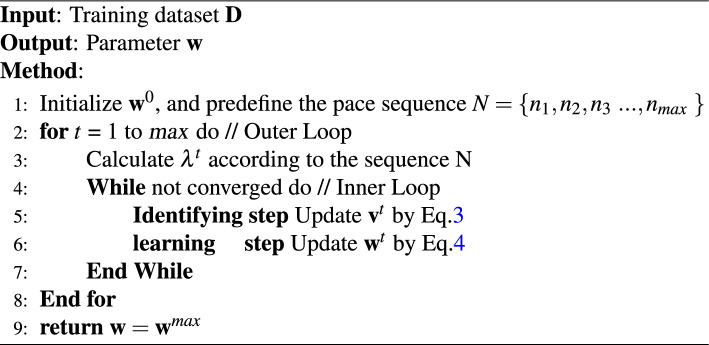
Self-paced Graph Neural Network (SPGMN).

#### Data driven multiple evaluation

In this paragraph, we will demonstrate how to use the SPGMN model to accomplish the distinctive student detection (DSD) task and use its results as a supplement to GPA assessment. The details of the method are outlined in Algorithm 2. The output results of Algorithm 2, SPGMN-DSD (distinctive student detection based on SPGMN), can be used to explain several phenomena that cannot be explained within the GPA evaluation framework: 1) Students with different learning abilities achieve the same grades; 2) Students with the same ability obtain significantly different scores. Additionally, these results can be utilized to identify exceptionally talented students (who achieve high grades with minimal practice) and students in urgent need of assistance (who have poor performance despite extensive practice). These conclusions mentioned above will greatly assist in the development of personalized learning and have also been validated in our subsequent experiments.

As discussed earlier, outliers exist in the dataset $\textbf{D}$, which are representative of distinctive students. We aim to identify these distinctive students by detecting outliers, specifically samples with larger training losses. Thus, in the initial stage, we train an SPGMN model for the SLPP task using the training data $\textbf{D}$. After a sufficient number of iterations, we obtain a trained model as well as a training loss list $\textbf{L} = \{\ll _{1},...,\ell _{n}\}$, where $\ell _{i}$ represents the training loss of the *i*th sample. Notably, the list of losses for distinctive students (outliers) differs from the set of losses for common students (majority of training samples). To exploit this distinction, we first sort the $\alpha$ values, which are obtained by dividing the loss of a target student by the sum of all losses. Subsequently, we randomly select a breakpoint from the list of non-zero $\alpha$ values and create two series of $\alpha$ values. Finally, if these two series fail to satisfy the t-test, we identify the students corresponding to the series with larger $\alpha$ values as distinctive students. The detailed steps are provided in Algorithm 1. Additionally, we repeat the SPGMN-ASD algorithm 400 times and consider the *k* students with the highest frequency of appearance as distinctive students, where *k* is the average of the 400 experimental results.

**Algorithm 2:**
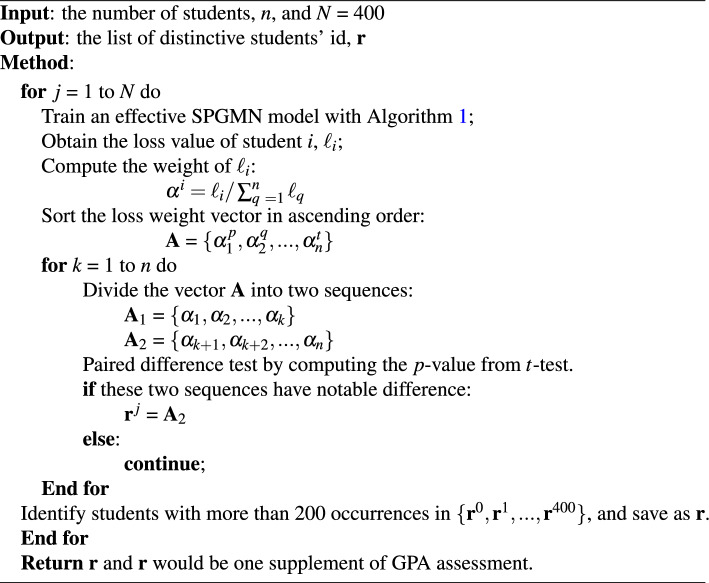
Distinctive Student Detection Based on SPGMN (SPGMN-DSD).

### Theoretical analysis

Moreover, in this section, we conduct a theoretical analysis to evaluate the robustness of the proposed SPGMN model. For the sake of clarity, we denote $\ell (y,g(\textbf{x}, \textbf{w}))$ as $\ell (\textbf{w})/\ell$ in the following discussion. The optimization strategy employed by SPGMN strictly adheres to a majorization-minimization algorithm, implemented on a latent objective. The loss function embedded in this latent objective bears similarities to a non-convex regularized penalty^31,32^. Considering this, we derive the optimal solution for $\textbf{v}^*$ in the SPGMN optimization process

6 $$\begin{aligned}  \textbf{v}^*(\ell ,\lambda ^k)\ = \underset{\textbf{v}}{{\text {argmin}}}\textbf{v}\ell \ + \lambda ^k \left( \frac{1}{2} \textbf{v}^{2}-\textbf{v}\right) . \end{aligned}$$

It is worth noting that when $\lambda ^k = \infty$, the latent loss $F_{\lambda ^k}(\ell )$ reduces to the original loss function $\ell$. Notably, $F_{\lambda ^k}(\ell )$ exhibits a pronounced suppression effect on large losses compared to the original loss function $\ell$. Once $\ell$ surpasses a specific threshold, $F_{\lambda ^k}(\ell )$ becomes a constant. This phenomenon offers a rational explanation for the robust performance of SPGMN even in the presence of outliers. Samples with loss values exceeding the threshold do not influence model training due to their gradient being zero. As a result, SPGMN avoids incorporating noisy information from outliers during the learning process.

The integrative function of $\textbf{v}^*(\ell ,\lambda ^k)$ can be calculated by Eq. (6) as:

7 $$\begin{aligned}  F_{\lambda ^k}(\ell )\ =\ \int _{0}^{\ell }{\textbf{v}^*(\ell ,\lambda ^k)\ dl} + c, \end{aligned}$$

where c is a constant. Here, we focus on the calculation result of Eq. (7) :

8 $$\begin{aligned}  F_{\lambda ^{k}}(\ell )=\left\{ \begin{array}{c} -\frac{1}{2 \lambda ^{k}} \ell ^{2}+\ell +c, \ell \le \lambda ^{k} \\ c, \ell \ge \lambda ^{k} \end{array}\right. \end{aligned}$$

## Experiments

In this section, we first validate the effectiveness of our improved Graph Memory Networks (GMN) model on two public datasets, namely Enzymes^33^ and Collab^17^. Specifically, we will validate the effectiveness of introducing self-paced learning (SPL) into GMN using these two datasets. Next, we design an experiment to evaluate the improvement of the proposed Self-Paced Graph Memory Networks (SPGMN) using the OULAD and NPU-GPA datasets to predict student learning performance (SLPP). For the task of distinctive student detection (DSD), we aim to uncover novel insights that cannot be captured by the GPA metric alone using the NPU-GPA and OULAD datasets. The statistical information in Table 1 indicates that our datasets are balanced.

The datasets are summarized and described as follows:

The *Enzymes* dataset is designed for the prediction of functional classes of enzymes. It consists of 600 graphs representing enzymes, each belonging to one of six categories.

The *Collab* dataset aims to predict the field of a researcher based on their ego collaboration graph. It contains 5000 graphs, which are divided into three classes.

The *NPU-GPA* dataset is used for predicting the final GPAs of students based on their course history scores. It includes 600 students from the School of Computer Science at Northwestern Polytechnical University. The students in the NPU-GPA dataset are divided into four categories based on their GPAs, specifically, GPAs ranging from 1 to 4.

The *OULAD* dataset stands for the Open University Learning Analytics dataset. It provides information about courses, students, and their interactions with the Virtual Learning Environment (VLE) for seven selected courses, known as modules. For our study, we obtained a smaller dataset containing 538 students who enrolled in one particular course. This course includes 5 tests and one final examination. The 538 students in this dataset are classified into three categories based on their final examination scores, namely, fail, pass, and distinction.

**Table 1:** Statistics information of datasets..

Datasets		
Label	NPU-GPA	OULAD
*Num*		
1	150	178
2	150	180
3	150	180
4	150	–

**Table 2:** Mean validation accuracy over 10-folds.

Method	Dataset	
	Enzymes	Collab
GMN	78.66	80.18
WL optimal	60.13	80.74
SPGMN	81.00	83.56

### Verification experiment

To evaluate the performance of SPGMN, we compare our method with the original GMN^15^ and the graph kernel method, WL Optimal Assignment^16^ on Enzymes and Collab datasets. Here, we follow the experimental protocol in^15^ and perform 10-fold cross-validation and report the mean accuracy of overall folds.

It is important to note that the results of GMN and WL Optimal Assignment reported in the original GMN research^15^ were adopted. We observed that WL Optimal Assignment outperformed GMN on the Collab dataset due to the presence of dense sub-graphs, which GMN is not effective in extracting near-optimal subgraphs from. However, in our experiments, our proposed SPGMN achieved better results than WL Optimal Assignment.

The results shown in Table 2 indicate the following: (1) Our proposed model, SPGMN, achieved better results compared to the GMN model, improving the performance on the Enzymes and Collab datasets by absolute margins of 2.34% and 3.38%, respectively. (2) In our verification experiment, SPGMN performed better than WL Optimal Assignment by an absolute margin of 2.82%. These findings indicate that SPGMN enhances the robustness of GMN and achieves improved prediction performance, which means that our method does effectively improve the performance of the original GMN.

**Figure 4:**
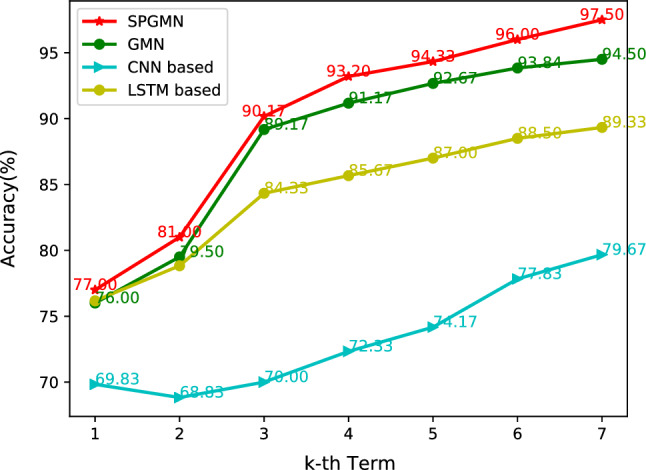
Mean validation accuracy over 10-folds.

**Table 3:** Mean validation accuracy over 10-folds. Note, GPA-*k* means the data of first *k* terms of GPA-data.

Methods	Datasets						
	NPU-GPA1	NPU-GPA2	NPU-GPA3	NPU-GPA4	NPU-GPA5	NPU-GPA6	NPU-GPA7
KNN	44.83	42.83	40.17	45.00	40.67	36.83	37.50
DTs	49.83	50.50	59.50	59.83	60.67	61.00	60.33
NBs	35.17	40.83	55.83	54.17	51.00	50.50	50.33
CNN	69.83	68.83	70.00	72.33	74.17	77.83	79.67
LSTM	76.17	78.83	84.33	85.67	87.00	88.50	89.33
GMN	76.00	79.50	89.17	91.17	92.67	93.84	94.50
SPGMN	77.00	81.00	90.17	93.20	94.33	96.00	97.50

**Table 4:** Mean validation accuracy over 10-folds. Note, OULAD-*k* means the data of first *k* test of OULAD. And, OULAD-6 means the data of 5 tests and the final exam.

Methods	Datasets					
	OULAD-1	OULAD-2	OULAD-3	OULAD-4	OULAD-5	OULAD-6
CNN	40.33	51.83	62.00	66.33	74.17	75.83
LSTM	48.25	62.15	69.64	74.57	80.78	81.16
GMN	52.04	64.34	72.64	77.57	83.59	84.96
SPGMN	53.26	66.23	75.47	79.62	85.29	86.85

### Student learning performance prediction experiment

To evaluate the improvement of proposed self-paced graph memory network (SPGMN) in student learning performance prediction (SLPP) task, we compared our SPGMN with the following baseline/SLPP methods on GPA-data: graph memory network (GMN)^15^, convolutional neural network (CNN)^11^, Long Short-Term Memory(LSTM)^34^ K-Nearest Neighborsna (KNN)^9^, decision trees (DTs)^35^, Naive Bayes (NBs)^10^. In the following experiments, SPGMN and GMN share one set of parameters. At the same time, other compared methods obtain its best parameters, especially in the SPGMN model, the pace sequence $N = \{16, 32,...,528, 540\}$ and $\lambda$ is calculated by Eq. (5) accordingly.

#### NPU-GPA dataset

In this study, a total of 600 students from four classes were analyzed using graph construction. In this graph, the nodes represent the courses that the corresponding students have enrolled in, and the top 5 related courses (determined by Euclidean distance) are connected with non-weighted edges. The objective is to predict the final GPAs (the GPAs after the last term) based on the data from the first *k* terms, where *k* is within the range of 1 to 7. The results of the SLPP task are presented in Table 3, while the trend of the prediction results is shown in Fig. 4.

From the analysis of Table 3 and Fig. 4, several important observations can be made. Firstly, our SPGMN model consistently outperforms other compared methods in all semesters, particularly the traditional prediction algorithm. Secondly, the advantage of the SPGMN model becomes more pronounced as the semesters progress. This could be attributed to the fact that as more samples are included in the training dataset, the trained model encounters more complex samples. However, the SPGMN model effectively reduces the number of transitions within these complex samples. Thirdly, it is worth to note that the curve between the second term and third semester shows a sharp rise compared to other stages, indicating that the third term is crucial for obtaining a better GPA. This can be explained by the fact that students require time to adapt to the new environment and develop effective learning strategies in the first two terms. Additionally, more courses are available in the third term, which could contribute to better academic performance. Lastly, the accuracy of KNN drops from 44.83% in the first term to 37.50% in the last term. This decline can be attributed to the increased data dimensionality caused by taking more courses, leading to issues with dimensional disasters. Following Jerome Bruner’s spiral curriculum idea, students’ comprehension improves over time with ongoing teaching. As a result, their GPA and grades may fluctuate as they progress through the curriculum.

#### OULAD dataset

During a selected course, a total of 538 students interacted with the Virtual Learning Environment (VLE). We logged the daily interactions of all students, categorizing them into 11 different types based on the learning material they clicked on. Additionally, we recorded the scores of all 5 tests and one final examination. To reconstruct the learning process as accurately as possible, we constructed interactive graphs using the data from 538 students. Each node in the graph represents one day’s interactive data and includes information about the corresponding test. The top 5 most similar nodes, as determined by Euclidean distance, are connected to each node through non-weighted edges.

The results displayed in Table 4 demonstrate that our proposed SPGMN method outperforms the compared methods. Both the NPU-GPA and OULAD datasets yielded experimental results indicating that the SPGMN method performs better than the original GMN algorithm. Consequently, we achieved a satisfactory outcome for the subsequent distinctive student detection (DSD) task.

### Distinctive students detection

This section presents our experimental approach for the task of distinctive student detection (DSD). In order to evaluate the performance of SPGMN-DSD (distinctive student detection based on SPGMN), we conducted two experiments using the NPU-GPA7 dataset and the OULAD dataset. In these experiments, our focus was on identifying the distinctive students solely within the training dataset.

#### NPU-GPA dataset

The NPU-GPA dataset, a proprietary dataset from X, comprises scores from all courses and registration information. However, detailed insights into the learning process remain inaccessible. In this section, we utilized the NPU-GPA7 dataset to identify distinctive students (DSs) within its 540 training samples.

**Figure 5:**
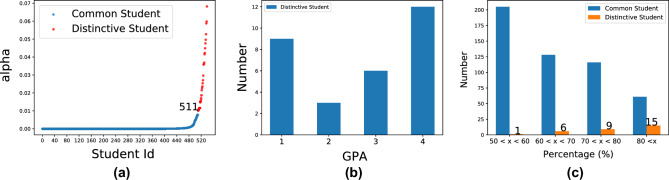
(**a**) Results of DSD task of NPU-GPA7; (**b**) Statistic of distinctive students; (**c**) The relationship between *x* and distinction. And, *x*is the percentage of major-related courses cut of all elective course.

Initially, a proficiently trained SPGMN model was derived from the SLPP experiment on the NPU-GPA7 dataset. Subsequently, distinctive students were identified among the 540 students. As depicted in Fig. 5a, the distinctive series (red) and the common series (blue) exhibit a significant gap at the 511th point, indicating that these two series originate from different distributions. Furthermore, Fig. 5b reveals that GPA alone is insufficient for student evaluation as distinctive students comprise individuals with varying GPAs.

**Figure 6:**
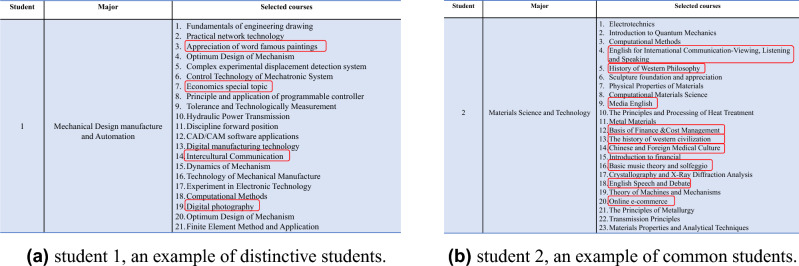
The example of elective courses that distinctive/common student has ever enrolled. Note, that these courses do not include required courses that students must enroll in during university..

Upon studying the 30 outliers, it was discovered that they do not represent errors but rather signify a notable event: the course selection strategy and learning pattern of distinctive students differ from those of common students. Specifically, mandatory courses in which students are required to enroll were excluded. As shown in Fig. 5c, common students (blue bars) tend to select non-major-related courses, such as the *Basics of Finance* for materials science and technology students. In contrast, distinctive students (orange bars) prefer major-related courses, such as *Database* for computer science students. When students enroll in more major-related courses, their learning patterns become more distinctive. Furthermore, Fig. 6 presents examples of the course selection of distinctive and common students, which are used to further validate the findings. The courses highlighted in red boxes are unrelated to the major and are general education courses. These observations suggest that learning patterns can complement GPAs evaluation framework: our method identifies individuals with a low GPA due to taking too many specialized elective courses. We recommend these individuals to consider taking some general education courses. Additionally, other individuals with a low GPA may need to allocate more time to their studies or seek additional assistance from their teachers.

This finding is corroborated by studies conducted by Dweck et al.^36,37^. Students adopt diverse learning approaches. Those with a growth mindset believe in enhancing their talents through diligence and persistence and are more autonomous in course selection, choosing courses based on their needs irrespective of course difficulty. Conversely, students with a fixed mindset perceive only obstacles; they fear that failing to choose a course will lead to poor grades and prioritize external validation, thus opting for relatively easy courses or seeking advice from seniors on easy-to-score courses.

Universities aim to foster students with a growth mindset; however, an evaluation system solely based on GPA fails to achieve this objective. A single GPA assessment is inadequate for student evaluation; course selection strategy and learning pattern could serve as valuable supplements to the GPA metric. Students’ learning patterns reflect their motivation and attitude towards learning-factors deemed crucial in student assessment tasks according to related educational psychology research^37^.

#### OULAD dataset

In this section, we delve deeper into the characteristics of distinctive students to uncover intriguing insights.

**Figure 7:**
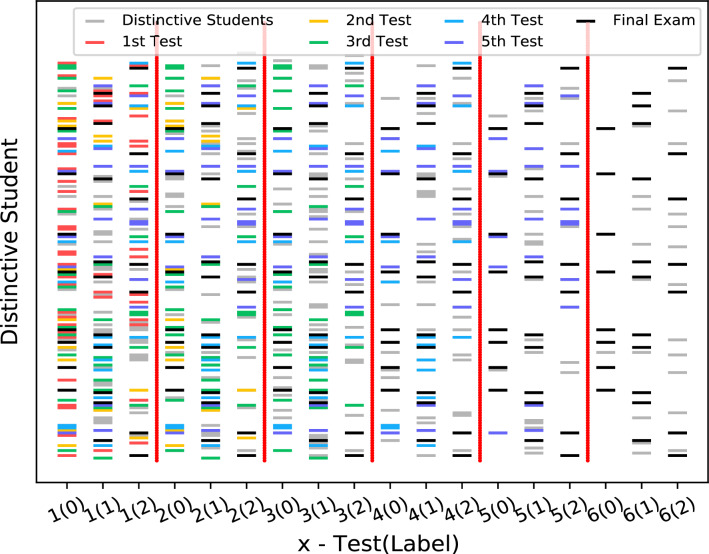
Distribution of distinctive students at different stages. And, the scale value of x-axis, i.e., *x*(*y*), is equal to *x*th test(GPA/label). All nodes in the diagram are distinctive students, and “*x*th Test” ($x \in \{1,2,3,4,5,6 \}$, and 6th test means the “Final Exam”) in the legend means that the students are distinctive until *x*th test.

The OULAD dataset, which comprises daily interaction data for all students, allows us to explore the intricacies of the learning process in greater detail. Initially, we utilized the trained SPGMN model from the SLPP experiment on the OULAD-*k* (k $\in$ {1,2,3,4,5,6}) dataset. Subsequently, we identified distinctive students within the OULAD-*k* training data. It’s important to note that not all students participated in all five tests or interacted with VLE. The OULAD dataset accurately captures the complex learning process of students, which can be convoluted. Consequently, as depicted in Fig. 7, the number of distinctive students (DSs) is expected to exceed the results from the previous distinctive student detection (DSD) experiment.

Figure 7 reveals that: (1) The number of distinctive students decreases from the first test to the final exam; (2) Distinctive students from the $(x+1)$th test are a subset of those from the previous *x*th test; (3) The numbers of distinctive students in the first three columns (i.e., $x \in \{1,2,3\}$) exceed those in the last three columns (i.e., $x \in \{4,5,6\}$). These observations lead us to conclude that: (1) Students’ learning status is initially varied due to diverse backgrounds but gradually converges to a steady state over time, as suggested by the plateau effect in educational psychology^38^. This results in a decreasing number of distinctive students; (2) Students’ learning state is typically continuous and does not change abruptly, a finding corroborated by prominent educational psychology research^39^; (3) We posit that most students are unprepared at the onset of a course.

**Figure 8:**
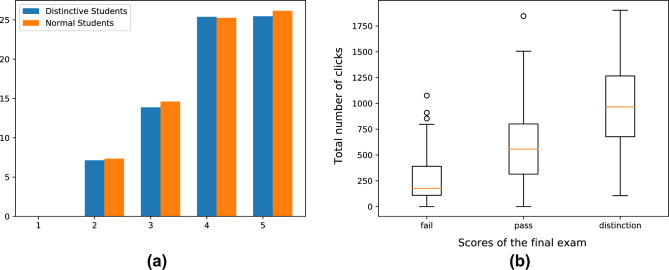
(**a**): Average weighted score of each test. Specially, the weights of 5 tests are [0,0.1,0.2,0.35,0.35] in order. (**b**): The boxplot of click number of students.

In OULAD, some students do not enroll in every test. Thus, in Fig. 8a, we first remove the recording of these students. Then, we compare the learning performance of common and distinctive students based on the remaining records. As shown in Fig. 8a, there is no significant difference in learning performance between common students and distinctive students. Combined with Fig. 7, we can conclude that SPGMN-DSD could mine more interesting facts than single GPA models, i.e., GPAs are not enough to evaluate students, and we need supplements of GPA.

**Figure 9:**
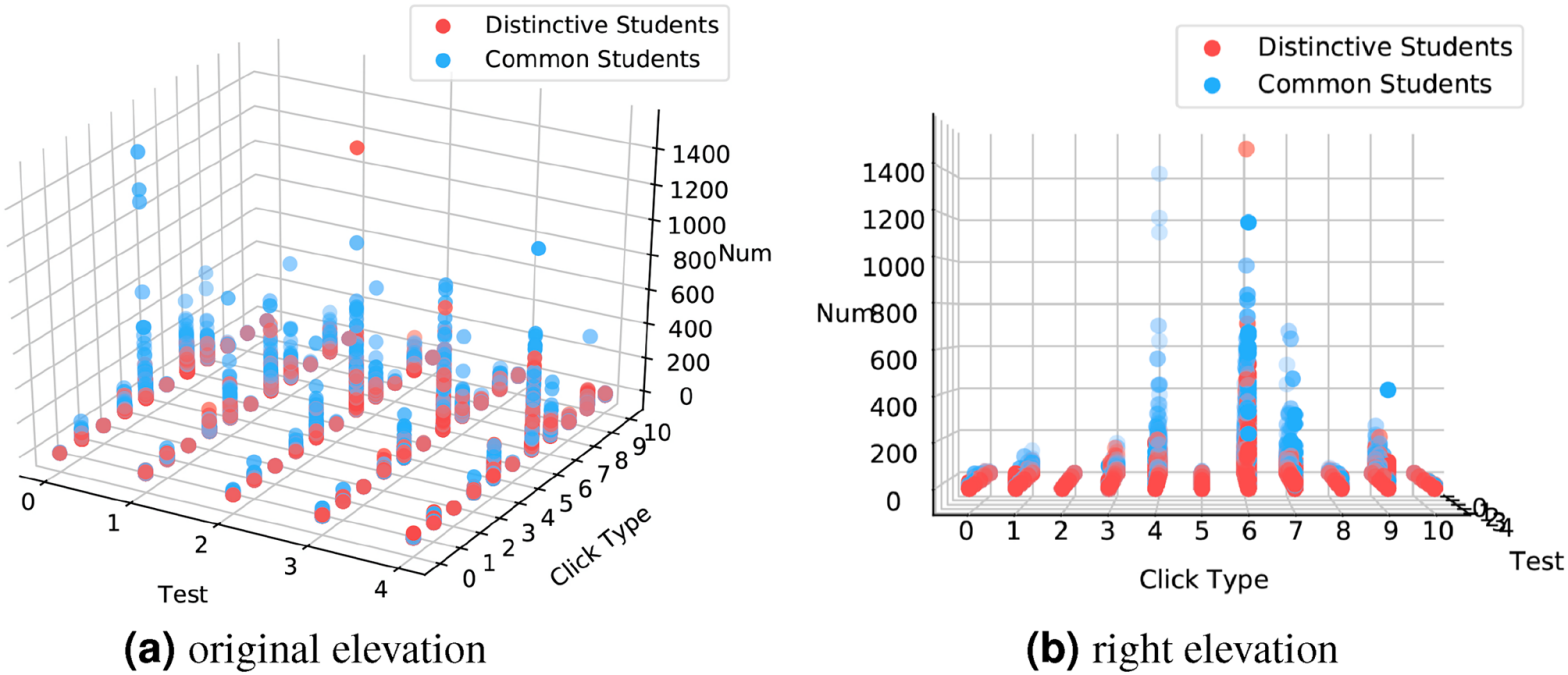
The click number of each students click on different online material.

Figure 7 provides intriguing insights into distinctive students, while Fig. 8a suggests that GPA alone is insufficient for student evaluation. Furthermore, Fig. 8b establishes a positive correlation between academic achievement and interaction frequency. Consequently, we aim to delve deeper into the factors influencing learning rewards by analyzing student interactions with the Virtual Learning Environment (VLE). Figure 9 presents a three-digit distribution map of student interactions with the online learning system. The cross-reference between online learning system materials and clicktype IDs is displayed in Table 5.

**Table 5:** Online material and clicktype cross-reference table.

Clicktype	0	1	2	3	4	5
Material	Oucollaborate	Subpage	Dataplus	Resource	Forumng	Url
Clicktype	6	7	8	9	10	
Material	Oucontent	Homepage	Glossary	Quiz	Questionnaire	

**Figure 10:**
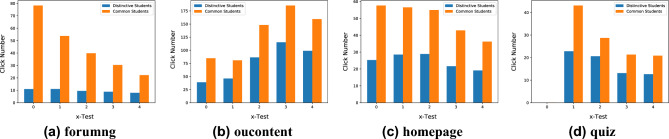
Average click number of each students click on selected online material.

As illustrated in Fig. 9a, the majority of the red dots are situated at the lower portion of the three-dimensional graph, while the blue dots are found at the top. From this, two key observations can be made: (1) There is a noticeable distinction between distinctive students and common students in their interaction with the online education system. Common students engage with the system more frequently and exhibit greater activity in the learning process compared to distinctive students. (2) The frequency of interaction with the online system has an impact on the outcome of the DSD task. Fig. 9b represents the right elevation and reveals that when $\textit{clicktype} \in$ {4,6,7,9}, which corresponds to the learning material categories of “forumng,” “oucontent,” “homepage,” and “quiz,” (as shown in Table 5) the blue dots have a wider distribution compared to other scenarios. This suggests that these specific learning materials have a significant influence on the outcome of the DSD task. In order to further investigate the impact of these factors, we extracted these corresponding data separately and generated individual graphs, as depicted in Fig. 10. From these figures, it is evident that common students are significantly more engaged in these aspects compared to distinctive students. Our approach effectively captures this observation, highlighting its ability to identify crucial information that is disregarded by the GPA evaluation framework. Among these distinctive students, gifted individuals exhibit proficiency in completing learning tasks independently without the need for extensive collaborative learning. Hence, these students should be provided with more challenging opportunities. On the other hand, other students, due to a lack of interest in learning or other reasons, engage less frequently with their classmates. Thus, it is crucial that we pay more attention to these students.

*Analysis* In this analysis, we will briefly discuss the potential impact of “forumng”, “oucontent”, “homepage”, and “quiz” on learning rewards. “Forumng” refers to the posts and discussions on the forum, while “oucontent” represents the various resources available on the Virtual Learning Environment (VLE), such as videos and powerpoints. The “homepage” denotes the front page of the VLE, which records the frequency of students logging into the system. Finally, the “quiz” records students’ self-regulation activities.

Based on the findings presented above, several conclusions can be drawn. First, GPA alone is insufficient for evaluating students. Second, the course selection reflects students’ motivation and attitude towards learning, which subsequently impacts their learning rewards. Third, the interaction between students and the system provides valuable insights into their learning status and influences their learning outcomes. Lastly, course selection and interaction are important aspects of the learning process that should be considered when evaluating students. Thus, it is crucial to incorporate quantitative indicators, such as course selection and interaction, when assessing student performance.

## Conclusion and future work

Student learning performance prediction (SLPP) is a fundamental aspect of assessing student performance and implementing personalized education. Unfortunately, most research primarily concentrates on predicting GPA with little consideration for identifying and analyzing distinctive students who do not fit the GPA models. Unfortunately, most research primarily concentrates on predicting GPA with little consideration for identifying and analyzing distinctive students who do not fit the GPA models. These students possess a wealth of informative knowledge that is invaluable in guiding further research on personalized learning. Based on this consideration, we have developed a data-driven student multi-dimensional evaluation framework as a supplement to the GPA evaluation framework, and it is used to address the deficiencies of the GPA framework.

In the first step, we introduced the self-paced learning (SPL) framework into the graph memory network (GMN) model to enhance its representation ability during the SLPP task. Subsequently, a well-trained SPGMN model was utilized for the distinctive student detection (DSD) task, resulting in the SPGMN-DSD (distinctive student detection based on SPGMN) model. The loss list of 540 training samples was divided into two series based on a t-test, namely distinctive students and common students. Moreover, we verified the influence of the course selection strategy and the interaction between the learning system and students on the outcomes of the DSD task and student assessments. By considering these factors, we aimed to explain phenomena that are not accounted for by the two GPA evaluation frameworks. These include situations where students with varying learning abilities achieve similar GPAs or where students with similar abilities receive significantly different GPAs. Additionally, these findings have the potential to identify exceptionally gifted students and those in need of immediate assistance. Ultimately, they contribute to enhancing the reliability and scientific nature of the GPA assessment framework, thereby offering substantial support for the development of personalized education.

However, this article has certain limitations. We acknowledge the importance of educational experiments in further validating the findings of our study. We understand that these experiments can provide more concrete evidence and insights into the topic. Unfortunately, due to various constraints such as time, resources, and ethical considerations, we were unable to conduct educational experiments in this particular study. In our future endeavors, we will allocate two academic years to design educational experiments in authentic educational settings (both online and offline) with the intention of validating our findings and augmenting our research.

## Data Availability

The datasets generated and/or analyzed during the current study are available in the project of Open University Learning Analytics dataset^40^, you can download the dataset from https://analyse.kmi.open.ac.uk/open_dataset.
